# Case Report: A novel chemotherapy-free regimen combined with photodynamic therapy, target therapy, and immunotherapy in a geriatric male with huge recurrent scalp and facial angiosarcoma: a report of an extremely rare case and literature review

**DOI:** 10.3389/fimmu.2025.1556493

**Published:** 2025-04-29

**Authors:** Ewetse Paul Maswikiti, Zedong Feng, Zhenyu Yin, Fan Zhang, Lijuan He, Baohong Gu, Lin Xiang, Huixia Li, Caijuan Wang, Yang Yu, Bo Xu, Jize Wang, Hao Chen

**Affiliations:** ^1^ Surgical Oncology Department, Lanzhou University Second Hospital, Lanzhou, China; ^2^ Department of Pathology, Lanzhou University Second Hospital, Lanzhou, China; ^3^ Department of Surgical Oncology, Lanzhou University Second Hospital, Lanzhou, China; ^4^ Key Laboratory of the Digestive System Tumors of Gansu Province, Lanzhou, China

**Keywords:** chemotherapy-free, photodynamic therapy, immunotherapy, target therapy, scalp and facial angiosarcomas, recurrent, geriatric patient

## Abstract

Angiosarcomas are sporadic vascular neoplasms among the most aggressive subtypes of soft tissue sarcomas. In addition, vast and multiple recurrent superficial scalp and facial angiosarcomas are very complex and extremely difficult to manage. Their occurrence brings about significant social and emotional distress to affected individuals. To date, no specific therapeutic strategy has been the most effective and reliable. Herein, we report a highly unique case of a geriatric male patient with recurrent scalp and facial angiosarcoma successfully treated by a chemotherapy-free regimen consisting of photodynamic therapy (PDT), immunotherapy, and target therapy. Notably, PDT provided promising remarkable auspicious outcomes and proved to be a better therapeutic option for refractory malignant angiosarcomas.

## Highlights

- Angiosarcomas are sporadic vascular neoplasms among the most aggressive subtypes of soft tissue sarcomas affecting the geriatric population.
- In addition, vast and multiple recurrent superficial scalp and facial angiosarcomas are very complex and extremely difficult to manage.
- A chemotherapy-free regimen combined with photodynamic therapy (PDT), immunotherapy, and target therapy has proven to be a better therapeutic strategy for managing facial aggressive angiosarcomas.
- Only a single case was treated and resulted in auspicious outcomes.

## 1 Introduction

Angiosarcomas (AS), also known as “malignant angioendothelioma”, are extremely rare, clinically aggressive tumors with limited treatment options with some dismal, thorny prognosis (1). AS contributes to approximately less than 2% of all soft tissue sarcomas in the human population and is commonly found in the geriatric population (2). AS is an infiltrative tumor with a high local recurrence rate and readily metastasizes (1). The etiology of AS is still unknown. What is known so far is that AS can arise from almost any body part, with cutaneous angiosarcomas (CAS) being the most common. In addition, CAS of head and neck lesions accounts for approximately 60% of AS cases (3). Some case reports and related studies have shown that CAS of the head and neck is a complex disease to manage and control, with a 5-year survival rate ranging from 10 to 54% (4–6).

There is no consensus amongst clinicians and dermatologists as far as its treatment options are concerned. However, there is vivid evidence that surgery and chemotherapy are the most effective treatment modalities in the management of AS (7). Surgical resection is guaranteed to be an essential component of the treatment strategy for AS whenever possible. Adjuvant radiation therapy after maximal surgical resection is commonly administered to improve local control. Nonetheless, CAS of the head and neck region may involve the skin extensively with multifocal lesions, making it difficult to acquire a wide negative margin despite the attempt for radical resection. The rate of local recurrence after surgery alone for AS escalates to 75% (8). Chemotherapeutic agents with activity against AS mainly include anthracyclines and taxanes. However, the role of adjuvant chemotherapy is not well defined. No prospective randomized studies have been conducted to provide treatment guidelines. Despite the availability of multimodal treatment, the recurrence remains high even for localized tumors (9).

Recently, photodynamic therapy (PDT) has been considered a more profitable, beneficial, and effective therapeutic strategy for various cancers (10, 11), but very few reports regarding its management in AS. In regard to the PDT strategy and process, irradiated tumors with specific wavelengths can activate photosensitizers that selectively aggregate in tumor tissues and trigger photochemical reactions. Furthermore, PDT oxidizes intracellular molecules such as proteins and lipids in tumor cells by generating active, reactive oxygen species (ROS), which leads to the damage, destruction, and apoptosis of tumor cells. In addition, some studies (10, 11) from our research group have found some synergy between target therapy and immunotherapy after PDT in patients with malignant tumors.

Regarding the management of scalp and facial AS, no similar studies, clinical work, or searched literature have ever reported a novel chemotherapy-free regimen combined with photodynamic therapy, target therapy, and immunotherapy. Herein, we report a 73-year-old male patient with a huge recurrent malignant scalp and facial angiosarcoma who underwent surgical resection and different types of chemotherapy at other medical institutions but developed persistent disease recurrence and progression. However, after utilizing photodynamic therapy, immunotherapy, and target therapy in our health center, the patient achieved a complete response (CR) in three months and had no signs of recurrence after one year of follow-up.

## 2 Case report

### 2.1 Chief complaints

A 73-year-old male patient presented to the authors’ health center, complaining of multiple bulging-type swellings on the forehead and behind the ears on both sides of the head, with surface ulceration and bloody fluid exudation for more than nine months post-surgery after having been confirmed with a clinical diagnosis of angiosarcoma.

### 2.2 History of presenting illness

**Figure 1:**
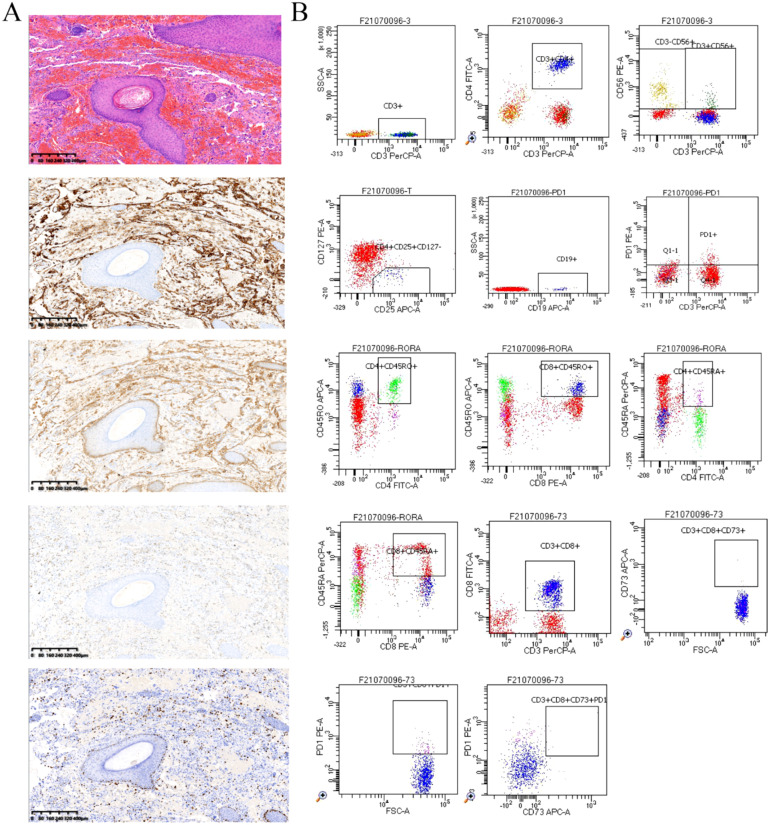
**(A)** Scalp hemangiosarcoma specimen (4×, by K-Viewer), post-operative pathological findings Hematoxylin and eosin (HE) staining and Immunohistochemical staining of CD31(+), ERG (+), D2-40(+), Ki-67 (20%). **(B)** Detection of peripheral blood immunological evaluation by a flow cytometric technique.

This male client was diagnosed with scalp and facial angiosarcoma at Xijing Hospital ten months before admission. He underwent “angiosarcoma resection” and “free flap repair”. His surgical specimen’s volume measured 13×12×2cm^3^, with irregular borders and a grey-red resurface with hair attachment. Pathological findings (**Figure 1A**) revealed an angiosarcoma with CD31 (+), CD34 (+), D2-40 (+), ERG (+), Fli-1 (+), FosB (+), Glut-1 (+), Ki-67 (20%), SMA (-), AE1/AE3 (-).

He was then discharged after very good signs of wound healing and union. Postoperatively, he declined radiotherapy and underwent nine cycles of chemotherapy at Gansu Provincial People’s Hospital three months later. Firstly, he was initialized on two cycles of a dual combination chemotherapeutic regimen comprising pirarubicin (60mg, iv, d1) and ifosfamide (2.5mg, iv, d1 to d5). Then the chemotherapeutic agents were switched to paclitaxel (275mg, iv, d1) for one cycle and albumin-bound paclitaxel (450mg, iv, d1) for three cycles because of the worsening disease condition. Notably, the patient also underwent “transnasopharyngeal radioactive particle implantation” while on chemotherapeutic regimens. After that, he continued two cycles of albumin-bound paclitaxel (450mg, iv, d1). However, there was no significant improvement. Finally, the patient underwent one cycle of gemcitabine (1.4g, iv, d1; 1.2g, iv, d5) instead of albumin-bound paclitaxel. Unfortunately, all nine cycles of chemotherapeutic regimens and radioactive particle implantation resulted in an extremely worse progressive disease (PD). During chemotherapeutic drug administration, he also developed generalized itching and numbness of the feet and soles, which led to some difficulties in ambulation. With the signs above, he discontinued and terminated treatment resulting in him being discharged from the hospital.

### 2.3 Physical examination

On physical examination multiple bulging type swellings on his forehead and behind the ears on both lateral sides of the head, a huge one measuring approximately 10×3×3cm^3^, with surface ulceration and bloody fluid exudation were noted. Nonetheless, no abnormalities were observed and detected on the examination of the chest, abdomen, and both upper and lower extremities.

### 2.4 Laboratory test findings

According to some laboratory test findings, the patient’s abnormal results were as follows (**Table 1**): RBC 3.94×10^12^/L (4.30-5.80×10^12^/L), PLT 111×10^9^/L (125-350×10^9^/L), Glu 6.96mmol/L (3.90-6.10mmol/L), CREA 51.9umol/L (57.0-111.0umol/L), LDH 311U/L (100-300U/L), TSH 4.808uIU/ml (0.380-4.340uIU/ml). Myocardial enzyme test findings were normal, and some test results of tumor markers: AFP, CEA, CA125, CA72-4, CA199, NSE, CY211, and SCC showed no significant abnormalities.

**Table 1:** Peripheral blood testing results.

Test Type	Test Item	Day 1	Day 38	Day 56	Day 76
Blood routine	WBC	6.38	3.70	4.89	8.06
	NEUT#	4.75	2.06	2.76	6.25
	RBC	3.94	3.78	4.69	3.94
	HGB	134	122	147	126
	PLT	111	138	157	102
Blood biochemistry	ALT	10	9	13	20
	AST	23	20	24	29
	DBIL	0.1	1.5	0.1	1.2
	IBIL	16.6	9.8	11.7	10
	TBIL	16.7	11.3	11.8	10.2
	TP	75.3	68.5	80.2	73.3
	ALB	42.3	34.4	39.5	38.36
	GLU	6.96	6.75	19.47	8.23
	TRIG	1.65	1.49	2.96	1.57
	TC	4.55	3.90	5.01	5.77
	CRE	51.9	67.3	60.5	71.1
	UA	418.0	345.0	327.0	358.9
Thyroid function	T3	1.51	1.20	1.53	–
	TSH	103.40	94.60	90.10	–
	FT3	4.49	4.15	5.36	–
	FT4	17.77	16.19	15.71	–
	TSH	4.808	9.972	14.579	–
Myocardial function	CKMB	1.75	–	–	1.85
	hs-cTnT	11.98	–	–	10.26
	MYO	42.65	–	–	24.86
	BNP	54.0	–	–	48
Coagulation function	PT	10.6	11.2	10.6	10.3
	PT-INR	0.96	1.02	0.96	0.94
	APTT	33.0	32.4	32.7	30.6
	Fib	3.85	3.51	3.72	3.98
	TT	13.4	14.8	13.3	13.9
	D-dimer	2.33	1.27	0.98	1.12

Blood routine test results: liver function, kidney function, heart function, thyroid function, and coagulation function tests in three treatment cycles.

**Figure 2:**
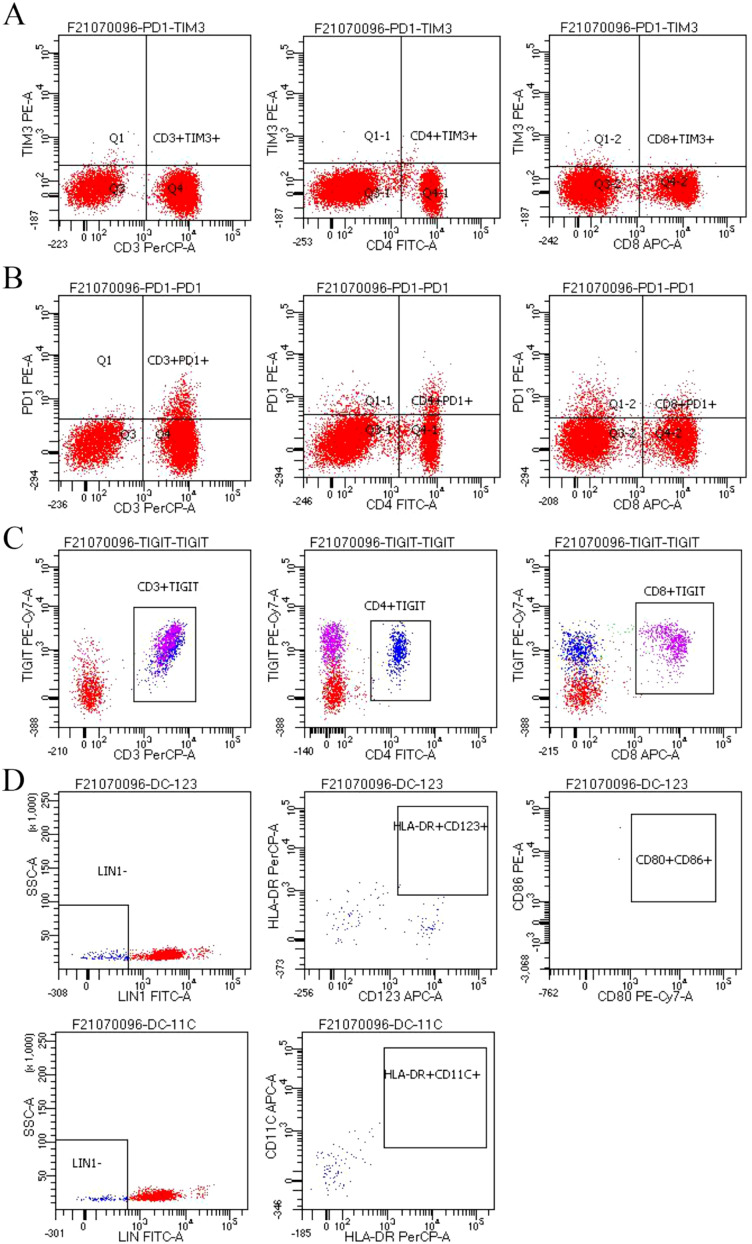
**(A-D)** Negative CTLA4+CD8 cells and positive LAG3+CD8, TIM3+CD8, PD-1+CD8, CD-CD19-CD14+CD16-HLA-DR cells were confirmed by a flow cytometric technique.

In addition, we performed some peripheral blood tests to evaluate the immunological functions (**Table 1**). Flow cytometry showed a decrease in proportion of LYMPH, CD19+, CD3+CD4+, CD4+CD45RA+, PD1+; a decrease in the amount of CD3+, CD19+, CD3+CD4+, CD4+CD45RA+, CD4+CD45RO+ cells (**Table 2**); an increase in the proportion of NEUT, and CD8+CD45RA (**Figure 1B**). The above just mentioned results implied that the patient’s immune function had been severely compromised and suppressed. In addition, immune checkpoint expressions were evaluated and are illustrated in (**Table 3**) The negative CTLA4+CD8 cells and positive LAG3+CD8, TIM3+CD8, PD-1+CD8, CD-CD19-CD14+CD16-HLA-DR cells were confirmed by a flow cytometric technique (**Figure 2**).

**Table 2:** Detection of peripheral blood immunological findings.

Content	Numeric value	Tips	Units	Reference range
WBC	6.62		10^9/L	4.00––10.00
%NEUT	77.7	↑	%	50.0––70.0
%LYMPH	14.1	↓	%	20.0––40.0
%MONO	4.8		%	3––12
#NEUT	5.15		10^9/L	2.00––7.00
#LYMPH	0.93		10^9/L	0.80––4.00
#MONO	0.32		10^9/L	0.12––1.2
CD3	66.5		%	50––84
CD19+	3.1	↓	%	5––18
CD3-CD56+	14.8		%	7––40
CD3+CD56+	4.5		%	3––8
CD3+CD4+	26.8	↓	%	27––51
CD3+CD8+	37.0		%	15––44
CD3+CD4+/CD3+CD8+	0.72			0.71––2.78
CD4+CD45RA+	4.0	↓	%	10––20
CD4+CD45RO	15.5		%	8––25
CD8+CD45RA	23.6	↑	%	10––20
CD8+CD45RO	14.4		%	2––15
PD1+	6.1	↓	%	10––16
CD4+CD25+CD127-	4.9		%	2––10
CD3+	618.45	↓	/ul	955––2860
CD19+	28.83	↓	/ul	90––560
CD3-CD56+	137.64	↓	/ul	150––1100
CD3+CD4+	249.24	↓	/ul	414––1123
CD3+CD8	344.10		/ul	238––874
CD4+CD45RA+	37.20	↓	/ul	154––485
CD4+CD45RO+	144.15	↓	/ul	298––683
CD8+CD45RA+	219.48		/ul	152––395
CD8+CD45RO+	133.92		/ul	85––261

↑, high; ↓, low.

**Table 3:** Immune checkpoint expression outcomes.

Content	Ratio of positive cells	Number of positive cells	Results	Reference range
CTLA4+CD8	0.030%	3	Negative	<10
LAG3+CD8	0.330%	33	Positive	<10
TIM3+CD8	0.140%	14	Positive	<10
PD-1+CD8	2.940%	294	Positive	<20
CD-CD19-CD14+CD16-HLA-DR	73.8%	/	Positive	<19.38%

### 2.5 Imaging examination studies

**Figure 3:**
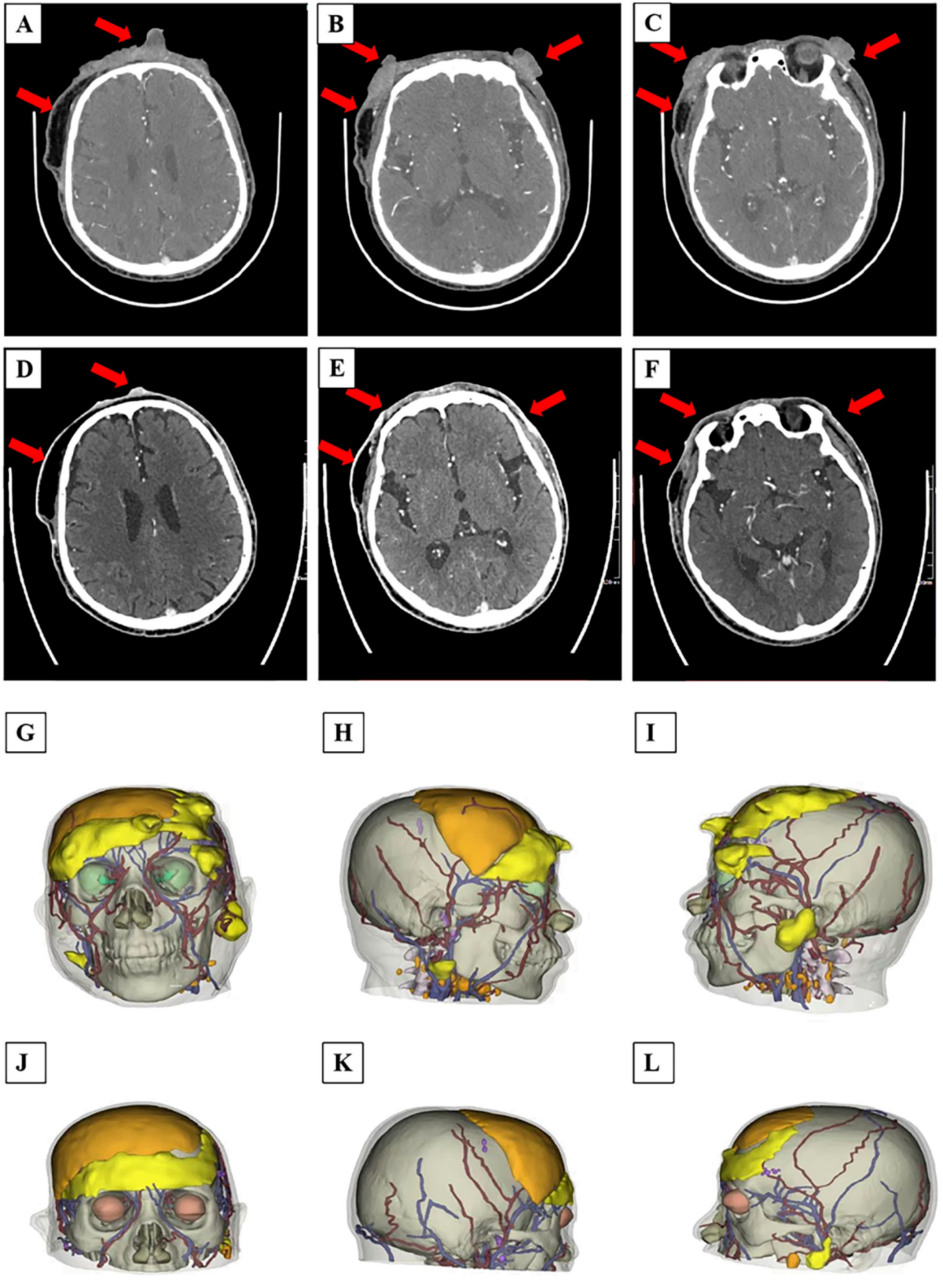
**(A–L)** CT scan images and 3D reconstruction printing of the patient before and after PDT treatment: **(A–C)** CT scan presented bilateral periorbital, bilateral frontal, left parietal, right temporal, and left mandibular paracortical subcutaneous soft tissue lesions of the patient after the first time PDT treatment; **(D–F)** CT scan showed some obvious and significant decrease in size of the recurrent malignant facial lesions before the second time PDT treatment. **(G–I)** 3D imaging of the patient’s tumor site before treatment, and **(J–L)** post-treatment. **(G, J)** the positive view. **(H, K)** the right view. **(I, L)** the left view.

An enhanced CT examination revealed these findings and 3D schematic images are in (**Figure 3**): 1. bilateral periorbital, bilateral frontal, left parietal, right temporal, and left mandibular paracortical subcutaneous soft tissue lesions, considered malignant lesions, consistent with the manifestation of scalp angiosarcoma; 2. postoperative manifestation of bilateral frontal and right temporal subcutaneous surgery, right frontal, parietal subcutaneous soft tissue swelling, right submandibular multiple enlarged lymph nodes; 3. right frontal bone, right parietal bone discontinuity, left occipital bone local depression, a right temporal arachnoid cyst.

### 2.6 Final diagnosis

The 73-year-old geriatric patient was diagnosed as having recurrent scalp and facial angiosarcoma with a KPS score of 60, an NRS score of 2, and an ECOG score of 1. The patient’s tumor stage was assessed as T_4_N_2_M_1_ according to the American Joint Committee on Cancer (AJCC), 2016.

### 2.7 The entire treatment process

After some in-depth discussions with the Multi-Disciplinary Team (MDT) members, it was decided that he be treated with a novel chemotherapy-free treatment regimen that comprised; PDT, target therapy, and immunotherapy (a trio therapeutic combination).

Ten days after admission, the patient received the first session of PDT at the author’s health center. Firstly, the patient was admitted into a non-light (dark cubicle, sideward) since PDT is a photophobic treatment procedure, wore light protective medical attire(clothing) and underwent a skin photosensitizer test in preparation for the PDT irradiation procedure for the first time, in which he tested negative. He was then intravenously injected with a photosensitizer, hematoporphyrin (Milelonge Biopharmaceutical Co., Ltd., China) of which its content was not fully examined at a dosage of 3mg/kg, 48 hours prior to his first irradiation PDT session. Notably, this agent (hematoporphyrin) commonly contains impurities including protoporphyrin and an analog where only one -C=C- group is present’.

Secondly, light irradiation of 633-nm at 1000 mW/cm^2^ via a laser therapeutic apparatus (PDT 630II laser photodynamic therapy instrument, XINGDA, China) at a total energy of 3600J was conducted 1cm away from the facial lesions for 60 minutes per every photodynamic therapeutic session he underwent. Collectively, for the entire PDT irradiation sessions we used 6cm laser fibers (XINGDA, China). The entire treatment process went on smoothly for four consecutive days, and the patient had no significant discomforts or abnormalities detected. Post-PDT, the patient was protected strictly from light and luminous sources for a month to prevent him from photoallergic adverse side effects. He could gain access to normal light a month later through some gradual light adaptive processes after a photo desensitization test was confirmed negative. Furthermore, 52 days after the first cycle of PDT, he was readmitted for a second session.

On the contrary, immunotherapy and target therapy were synchronized during the PDT irradiation sessions. He took immunotherapy, which was the administration of Tislelizumab (BeiGene Ltd., China), 200 mg, in a period of 21 days, and three cycles in total. Meanwhile, the patient was also on oral (PO) target therapy, Anlotinib tablets (Chia Tai Tianqing Pharmaceutical Co., Ltd., China), 12 mg daily for two weeks and had a week break in between, in also a period of 21 days, and three cycles in total.

Presently the patient is on oral (PO) Anlotinib for maintenance therapy.

### 2.8 Outcomes and follow-up results

**Figure 4:**
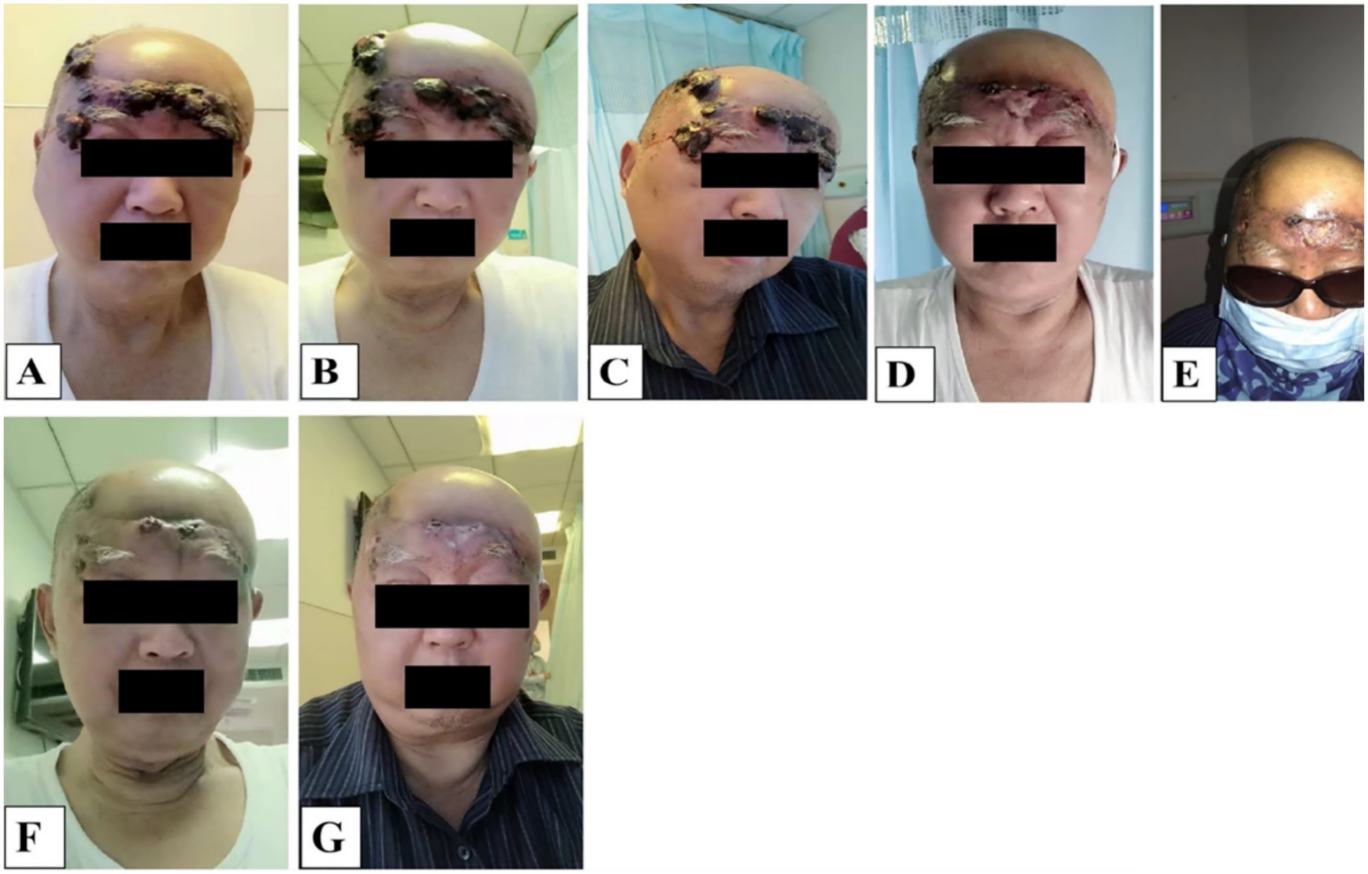
**(A-G)** The entire multiple recurrent angiosarcoma lesion morphological and appearance changes before and after PDT irradiation laser treatment. **(A)** the appearance of the tumor the first time on admission before PDT irradiation, **(B-E)** second visit just after having undergone PDT treatment, day 22, almost diminishing, **(F, G)** tumor appearance on day 44, last PDT irradiation, only scarring observed, tumor completely diminished and healed.

We also performed a 3D reconstruction printing technique for this patient for a more vivid tumor/lesion visualization before and after the patient’s treatment regimen, and the results showed that the patient’s tumor had a volume of 100.67 cm^3^ before and 27.45 cm^3–^49 days post the first treatment circle of PDT. Meanwhile, the patient’s massive tumor with severe bleeding (**Figure 4A**) from the lesion site gradually subsided (**Figures 4B–E**), and the wounds healed remarkably (**Figures 4F, G**). Combined with the patient’s enhanced CT and 3D reconstruction printing results (**Figure 3**), the patient’s treatment effect was regarded as partial response (PR) according to Response to Solid Tumors (RECIST) 1.1. By the end of the second session of PDT irradiation, wounds(lesions) gradually healed, and only some scar tissues were observed. Collectively, the patient’s treatment efficacy and remarkable outcomes were noted and regarded as complete resolution (CR) when evaluated by the naked eye.

Post-PDT irradiation sessions, combined with target therapy and immunotherapy, the patient assuredly had a tremendously good general condition and no significant adverse effects and abnormalities. Moreover, no significant abnormalities were detected after several tests in the patient’s blood glucose, liver and kidney function, thyroid function, cardiac function, and coagulation function (**Table 1**), which verified that the patient’s treatment process was safe and effective.

The patient has been followed up for more than one year with no abnormalities detected and remarkable auspicious outcomes.

## 3 Discussion

Angiosarcoma (AS) is an infiltrative tumor with a high local recurrence rate and readily metastasizes. Some studies have reported the metastatic rate of scalp and facial angiosarcomas at presentation to vary from 16 to 44%, and the overall survival (OS) rate ranging from 6 to 16 months (12). Despite the proliferation of studies regarding AS, the comprehensive treatment of AS remains a clinical challenge, especially for refractory and recurrent cases.

To date, only radical surgery performed at an earlier stage with negative margins can offer preferable prognostic outcomes in managing angiosarcomas (13). Moreover, regarding anatomical structures and the metastatic nature of angiosarcomas, most patients with angiosarcomas of the head and neck are not candidates for surgical resection. In addition, due to the difficulty in achieving a negative surgical margin, even after an extensive surgical approach, the rate of local recurrence and distant metastasis remains high (14). In regard to our case report, the patient underwent “angiosarcoma resection” and “free flap repair” from previous health centers. Notably, postoperative pathology results showed that tumor tissues were visible in the basal cut edge, which meant that he was likely to have a high recurrence and metastatic rate.

Theoretically, radical surgery followed by adjuvant radiotherapy has been considered a preferred current treatment modality. Some studies have demonstrated that patients who receive post-surgical radiotherapy have significantly improved survival rates compared to patients who receive surgery and radiotherapy alone (15). Conventional cytotoxic chemotherapy has been frequently used in treating inoperable and metastatic tumors. While there remain some controversies about systemic chemotherapy for angiosarcoma management, the general theory has been in agreement with the fact that adjuvant chemotherapy results in limited benefits to patients after surgery or radiotherapy. Collectively, primary chemotherapeutic agents included taxanes, doxorubicin, liposome doxorubicin, and ifosfamide. Paclitaxel has often been used in the first- or second-line treatment for angiosarcoma metastatic disease. A study (16) by R J Young et al. found that in 108 locally advanced and metastatic angiosarcoma patients, 25% of angiosarcoma patients had a complete or partial response under first-line anthracycline-based chemotherapy. In this case, the patient’s surgical procedure did not result in him undergoing radical resection, leading to a rapid disease recurrence because of the ineffective outcomes of chemotherapeutic drug administration. Of note, he received pirarubicin chemotherapy with poor results, and the ailment (angiosarcoma) continued to worsen after switching to gemcitabine.

In recent years, vascular endothelial growth factor (VEGF) and platelet-derived growth factor (PDGF) and their receptors have been the most significant angiogenic factors, the overexpression of which has been found in different subtypes of sarcomas, including angiosarcoma. Tyrosine kinase inhibitors (TKI) have been implemented in target therapy for managing angiosarcomas by inhibiting the VEGF/VEGFR signaling pathway. Furthermore, AS is derived and originates from vascular endothelial cells, and it has been reported that VEGF protein and VEGFR are overexpressed in 80% of all AS cases (17). On the contrary, Anlotinib is a novel small molecule, a multi-target tyrosine kinase inhibitor that effectively inhibits VEGFR, PDGFR, FGFR, c-Kit, and other kinases, and has anti-tumor angiogenic effects. Additionally, Ren B et al. reported a case of a 99-year-old patient with head and facial angiosarcoma who achieved partial remission after oral administration of Anlotinib (18). Contrarily, Weiran Xu et al. reported a case in which teraplizumab combined with anlotinib achieved PR outcomes in a 57-year-old patient with primary splenic hemangiosarcoma (19). Therefore, in this case, we used anlotinib as a target drug of choice.

Recently, it has been demonstrated that programmed death 1 (PD-1) and its receptors, including ligand-1 (PD-L1) and ligand-2 (PD-L2) are thought to be other effective therapeutic targets for malignant tumors. A certain study found that PD-1 and PD- L1 expression in 105 patients with multiple subtypes of sarcoma and found that intratumoral infiltration is PD-1 positive lymphocytes in 65% of cases and PD- L1 tumor expression in 58% of the cases (20). Furthermore, Vinod Ravi et al. collected data from 25 patients with angiosarcoma treated with pembrolizumab (a monoclonal antibody that binds to the PD-1 receptor) at MD Anderson Cancer Center and showed that the objective response rate was 18%, whereas the disease control rate was 59% (21). On the contrary, Simran Sindhu et al. reported a case of a 63-year-old patient with nasal angiosarcoma who developed liver metastases after surgery and chemotherapeutic administration. After 13 cycles of pembrolizumab treatment, the liver metastases were resolved (22). However, in this case, LAG3+CD8, TIM3+CD8, PD-1+CD8, CD-CD19-CD14+CD16-HLA-DR cells in peripheral blood were confirmed as positive by a flow cytometric technique, especially the positive PD-1+CD8, which further demonstrated that this patient potentially could have many benefits from immunotherapy.

The oldest literature search (23) on PDT for angiosarcoma dates back to 1978, when A Shiryaev et al. used hematoporphyrin as a photo synthesizer to achieve partial remission in patients with hemangiosarcoma under a red-light instrument. Patricia Soo-Ping Thong et al. (24) also reported a case of a 64-year-old Chinese male patient with a histologically proven multifocal angiosarcoma of the head and neck. When utilizing radiotherapy, followed by PDT, the patient’s distal arm metastatic tumor healed tremendously.

**Figure 5:**
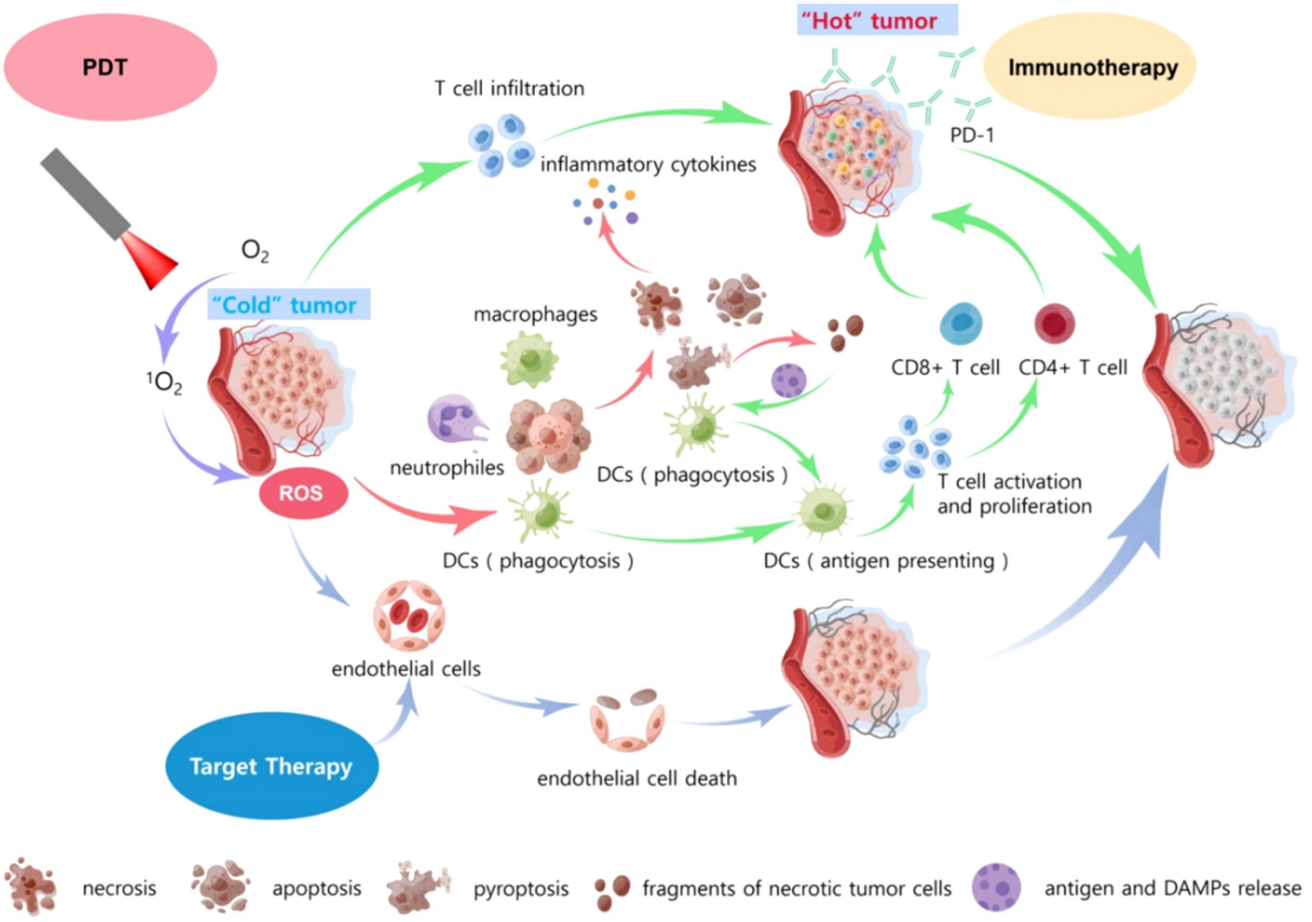
A schematic diagram of the synergistic effect of PDT, targeted therapy, and immunotherapy. Red arrows: under laser irradiation of PDT, O^2^ in the tumor microenvironment is converted to ^1^O_2_, generating several numbers of ROS to directly kill tumor cells. Then, neutrophils, macrophages, and dendritic cells are recruited into some numbers to surround the tumor cells, which undergo necrosis, apoptosis, and cell proptosis. Green arrows: The dead cell fragments and associated antigens, DAMPS, stimulate the DCs again, causing activation and dramatic proliferation of T-cells. A large infiltration of CD8+ T-cells and CD4+ T-cells then appears in the tumor tissue. Meanwhile, PDT causes T-cell infiltration. Based on the above, PD-1 inhibitors can better kill tumor cells. Blue arrows: ROS induced by PDT can destroy tumor endothelial tissue, and in combination with targeted therapeutic drugs, will cause massive death of tumor tissue endothelial cells, leading to hypoxic necrosis of tumor microvessels. In summary, the combination of PDT target therapy, and immunotherapy appears to be a very good synergy to achieve a better killing effect on tumor cells.

Presently, advantages and merits of PDT include (1) high selectivity, low traumatic outcomes, high applicability, repeatable treatment, no drug resistance, elimination of microscopic lesions, and preservation of vital organ function; (2) causation of a series of intracellular molecular signaling pathway changes and finally induction resulting in apoptosis, cytoplasmic lysis, organelle failure, and cell membrane rupture; (3) destruction of tumor-associated blood vessels, leading to tumor death due to hypoxia or nutrient deprivation; (4) derivation of a solid local immune response, promotion of a variety of proteasomes, peroxisomes, vasoactive substances, free radicals, coagulation cascade factors; (5) synergism with targeted therapies and immunotherapies (**Figure 5**).

Since the patient had already undergone anthracycline and taxane chemotherapy after surgery but yielded poor disheartening results and the aforementioned characteristics of angiosarcoma and new treatment modality, in our case, we finally selected and opted for target therapy, immunotherapy, and PDT in managing him.

Why does this patient benefit from PDT in combination with target therapy and immunotherapy? This must be figured out, the potential interaction of PDT, target therapy, and immunotherapy.

Gomer et al. (25) demonstrated that elevated expression of molecules such as VEGF, MMP, and COX-2 after PDT irradiation induces tumor neovascularization and regulates the tumor microenvironment. Therefore, PDT leads to the exposure of target molecules that promote tumor growth through the direct killing effect of tumor cells and the damage of vascular endothelial cells. Moreover, the application of target molecule inhibitors during this time can effectively slow down tumor progression. In tumors, and damaged endothelial cells, PDT causes a rapid and massive release of pro-inflammatory mediators and cytokines to help kill tumor cells, which means that PDT has some synergistic effects with chemotherapy and target therapy, reducing chemotherapeutic resistance to a certain extent. Reactive Oxygen Species (ROS) generated by PDT can cause platelet aggregation and vascular obstruction causing persistent tumor tissue hypoxic death, enhancing the effect of targeted therapy (26).

Although immunotherapy is considered a significant nemesis for multiple cancers, the response rate of immunotherapy only ranges from 10 to 40% (21). PDT can induce immunogenic cell death (ICD) (27) and strengthen the body’s innate and adaptive immunity by activating damage-associated molecular patterns (DAMPs). This process coincides and clashes with immunotherapy. It has been suggested that PDT can induce immunogenic enhancement of tumor T-cell infiltration and it is an effective immunogenic therapy that can sensitize tumors to immunotherapy and improve the tumor treatment efficacy. Collectively, some studies have revealed that a combination of immunotherapy with PDT significantly increases the response rate of tumors to immunotherapy.

## 4 Summary and conclusions

In summary, this case illustrated that PDT is associated with some significant clinical benefits for geriatric patients with recurrent scalp and facial angiosarcomas in combination with immunotherapeutic and targeted therapeutic agents. Most importantly, PDT is a promising therapeutic option for managing stubborn and aggressive solid malignant lesions even at an advanced late stage. Nonetheless, PDT irradiation therapy’s efficacy and safety are still to be elucidated, particularly if more cases such as ours could be enrolled in some large-scale clinical trials soon. However, there are some drawbacks and limitations to this work. (1) Due to the patient’s refusal and being uncooperative at some point in time during the treatment process, we lacked PD-L1 expression levels in tumor tissues, although we likewise found evidence in the peripheral blood that the patient could benefit from PD-1 inhibitors; (2) We did not detect changes in immune function in the peripheral blood after PDT combined with targeted therapy and immunotherapy.

## Data Availability

The datasets presented in this study can be found in online repositories. The names of the repository/repositories and accession number(s) can be found below: none.
